# A national cross-sectional study of patients with inherited retinal disease in China

**DOI:** 10.1038/s41598-026-70895-7

**Published:** 2026-09-09

**Authors:** Yudi Zhang, Mengyao Chen, Zhaoyan Duan, Panwang Yang, Liang Xie, Zhisheng Yang

**Affiliations:** 1 Department of Sociology, University of Science and Technology Beijing, Beijing, China; 2 Suzhou Aitong Vision Health Public Welfare Service Center, Suzhou, China

**Keywords:** Inherited retinal diseases, Health status, Living conditions, Cross-sectional study, China, Diseases, Health care, Medical research

## Abstract

To address the lack of comprehensive data on the health and living status of Chinese patients with inherited retinal diseases (IRDs). A questionnaire survey was conducted among 2218 IRD patients or their proxies, with data analyzed across six key dimensions: general features, family status, symptoms, diagnosis and treatment, rehabilitation, and life impact. The patients had a male-to-female ratio of ~ 6:4, mean age of 28.51 ± 17.14 years, 32.42% with disability certificates, and 27.68% of adults unemployed. Most (77.68%) had a per capita annual household income ≤ RMB 100,000. The mean age of onset was 13.11 years, with 50.09% reporting disease progression turning points (e.g., COVID-19, overwork). The average time from onset to diagnosis was 5.87 years, 42.47% experienced misdiagnosis, 33.81% received treatment (70.27% dissatisfied with effects), and economic capacity for treatment was limited but improved with long-term installments. Patients showed high willingness for rehabilitation but low acceptance of cross-city training; 45.27% used assistive devices, and 74.39% could travel independently, though IRDs severely impacted daily life. Chinese IRD patients face poor health and living conditions, including delayed diagnosis, high misdiagnosis, low treatment satisfaction, and economic constraints. Comprehensive social security policies are urgently needed to meet their multi-dimensional needs.

## Introduction

Inherited retinal diseases (IRDs) are a group of clinically and genetically heterogeneous diseases. Patients suffer from reduced visual function and even complete blindness resulting from photoreceptor degeneration or dysfunction^1^. The global prevalence of IRDs, including various subtypes, such as retinitis pigmentosa, cone–rod dystrophy, Leber congenital amaurosis, choroideremia, and X–linked retinoschisis, is approximately 1/1380^2^. Although IRDs are typically nonsyndromic (i.e., they entail only ocular involvement), a variety of syndromic IRDs also exist, the most common of which is Usher syndrome^3^. IRDs are inherited mainly on the basis of single genes, including autosomal dominant, autosomal recessive and X-linked inheritance^4^.

The common symptoms of IRDs include nyctalopia, a restricted visual field, difficulty in color discrimination, and difficulty adapting to changes in light level, in which nyctalopia is most common^5^. Clinical methods for treating IRDs have been very limited, and the main interventions have been genetic counseling, the use of visual aids and the treatment of complications, such as the installation of visual aids for patients with reduced visual acuity and the treatment of complications such as cataract and retinal neovascularization, but none of these measures can delay disease progression^6,7^. In recent years, retinal supplementation, stem cell transplantation, optogenetic therapy and retinal prosthetics have progressed as methods that do not rely on specific genes^8^. However, owing to the complexity of IRDs, the vast majority of patients still face treatment challenges^9^.

Owing to the lack of effective treatment, patients with IRDs suffer a progressive loss of visual function, which leads to reduced mobility and difficulties in reading, shopping, driving, and exercising on the part of patients^10–12^. After obvious vision damage occurs, patients with IRDs may become disoriented in familiar spaces, be afraid of crowded places, and experience difficulty performing small daily activities^13^. Given that IRD patients with severely impaired vision are limited in terms of abilities relevant to daily life and self-care, they often require formal or informal help from others^14^. In terms of employment, studies have shown that the unemployment rate of retinitis pigmentosa patients in the United States has reached 41.1%, which is significantly higher than the overall unemployment rate (3.8%) during the same period^12^. A survey in Japan^15^ also highlighted the difficulty experienced by IRD patients in the process of finding a job. The overall employment rate of retinitis pigmentosa patients was only 41.0%, which was far lower than the employment rate of the general Japanese population at the same age (82.2%). Not only do these individuals have difficulty finding employment, but patients with IRDs also face a severe economic burden of disease. Different studies conducted in the United States, Canada, the United Kingdom, Ireland, Singapore, and Japan have reported that the per capita annual cost faced by IRD patients is between US$6926 and US$275,045^14–17^. High disease expenditures make the lives of patients more difficult.

In summary, the health and living conditions of patients with IRDs deserves attention. Although several studies have investigated the health and living conditions of patients with IRDs, no large-scale survey has been conducted in China. On the basis of a prevalence of 1/1380, there are approximately 1 million IRD patients in China. The purpose of this study was to investigate their disease status, diagnosis and treatment history, and living conditions to obtain a comprehensive overview of the health and living conditions of IRD patients in China.

## Materials and methods

### Participants

This investigation was conducted with the help of an IRD patient organization. Registered in Jiangsu, China, this patient organization provides information consultation, builds an online patient communication platform, and promotes doctor–patient communication for IRD patients nationwide through channels such as official WeChat accounts and WeChat groups. The inclusion criterion used for this study focused on patients who were diagnosed with IRDs by a medical institution. The exclusion criteria were as follows: patients with ocular symptoms who had not been diagnosed with IRDs and patients who returned invalid questionnaires.

A total of 2218 valid questionnaires were received from 31 provincial administrative regions and the Hong Kong Special Administrative Region in China. A total of 969 questionnaires (43.69%) were self-completed by the patients or dictated by patients and filled out by others; 1249 questionnaires (56.31%) were completed by patient family members on the basis of the conditions of the patients and their families. The disease types of the patients were distributed as follows: retinitis pigmentosa (*N* = 1388, 62.58%), Leber congenital amaurosis (*N* = 177, 7.98%), Stargardt disease (*N* = 296, 13.35%), X- linked retinoschisis (*N* = 76, 3.43%), cone–rod dystrophy (*N* = 65, 2.93%), choroideremia (*N* = 29, 1.31%), syndromes (e.g., Usher syndrome and Bardet–Biedl syndrome) (*N* = 88, 3.97%), and other IRDs (*N* = 99, 4.46%).

### Questionnaire design

Four professionals with sociology or social work backgrounds and 2 IRD patients formed the questionnaire design team. The framework of the questionnaire was developed with reference to similar studies^18,19^. The first draft of the questionnaire was designed on the basis of interviews with 9 IRD patients and 11 family members and a review of the relevant literature, and was then optimized through multiple group discussions. Before the formal administration, we conducted a presurvey (*N* = 92). In the presurvey, participants did not report any problems with the structure or content of the questionnaire; however, three participants asked for better accessibility to make the questionnaire easier to complete. Based on this feedback, two improvements were made before the formal survey: (1) the survey was moved to an online platform with better accessibility (Tencent Questionnaire, wj.qq.com), which respondents can complete directly within WeChat—an application that passed China’s national accessibility evaluation for elderly and disabled users, offering a care mode with enlarged fonts and good compatibility with screen-reading software; and (2) respondents who had difficulty completing the online questionnaire because of visual impairment and had no one available to assist them could contact the research team and complete the survey by telephone. Finally, the medical part of the questionnaire was reviewed by an ophthalmic genetics specialist. The contents of the final questionnaire included general features, family status, symptoms, diagnosis and treatment, rehabilitation, and impact on life.

We did not adopt established instruments of visual function and vision-related quality of life, such as the 25-item National Eye Institute Visual Function Questionnaire (NEI-VFQ-25), for two reasons. First, the purpose of this study was to obtain a comprehensive overview of patients’ disease status, diagnosis and treatment history, and living conditions, rather than to measure vision-related quality of life or health utility; most of the constructs covered by the above sections fall outside the scope of these instruments, and survey studies of patients with rare diseases have likewise used study-specific questionnaires^18,19^. Second, the survey also included four psychological scales (the results of which will be reported separately), and the presurvey showed that the mean completion time already exceeded 25 min, as respondents with IRDs read and respond slowly due to impaired vision. Longer questionnaires are known to reduce respondents’ willingness to start and complete a survey and to degrade the quality of answers to questions positioned near the end^20^. We therefore used a concise, study-specific questionnaire.

### Procedure

In January 2025, the patient organization that cooperated with this survey recruited respondents through two channels: (1) the information of this survey was distributed on the WeChat official accounts of the patient organization, and (2) the survey posters were released in multiple WeChat groups of patients managed by the organization. The survey was performed from January to March 2025. The vast majority of respondents answered the questionnaire by scanning the quick response (QR) code contained in the recruitment information or clicking the link provided in that material. The research team conducted a telephone survey involving 20 respondents who had visual impairments, and no one could provide them with assistance.

### Data analysis

Data analysis was performed with the assistance of SPSS (Version 21). This study relied mainly on descriptive analysis. For continuous variables, the mean and standard deviation are reported; for categorical variables, the number and proportion of each category are reported. A *t* test was performed to analyze the difference between continuous variables, and a chi-square test was used to analyze the difference between categorical variables. A *P* value less than 0.05 indicated statistical significance. Microsoft Excel was used for visualization.

## Results

### General features

As shown in Table 1, among the 2218 patients in this study, 60.14% were male, and 39.86% were female; the age distribution was 28.51 ± 17.14 years. Adult patients (aged ≥ 18 years) accounted for 66.14% (*N* = 1467) of the respondents, and minor patients accounted for 33.86% (*N* = 751). Among adults, male patients accounted for 58.42% (*N* = 857) of the respondents, and female patients accounted for 41.58% (*N* = 610); among minors, male patients accounted for 63.52% (*N* = 477), and female patients accounted for 36.48% (*N* = 274). There was a significant difference in the sex ratio between adult and minor patients (*P* = 0.020). Among adult patients, 32.72% had never married. A total of 32.42% of patients held official disability certificates; in particular, 34.63% of adult patients and 28.10% of minor patients held disability certificates. According to data released by the Ministry of Civil Affairs of China in 2024^21^, there are more than 38 million people with disabilities who possess certificates in China, accounting for nearly 3% of the total population. The proportion of patients with documented disabilities in this study was much greater than that in the general population. A total of 27.68% of adult patients were unemployed, which is far higher than the urban unemployment rate of 5.1% in 2024^22^.

**Table 1:** General features of surveyed patients with IRDs.

	Category	Adults	Children	Overall
*N*		1467	751	2218
Age, mean ± SD		38.35 ± 12.23	9.28 ± 4.06	28.51 ± 17.14
Sex, *n* (%)	Male	857 (58.42)	477 (63.52)	1334 (60.14)
	Female	610 (41.58)	274 (36.48)	884 (39.86)
Ethnic group, *n* (%)	Han	1374 (93.66)	709 (94.41)	2083 (93.91)
	Other	93 (6.34)	42 (5.59)	135 (6.09)
Marriage, *n* (%)	Never married	480 (32.72)	751 (100.00)	1231 (55.50)
	Married	890 (60.67)	NA	890 (40.13)
	Divorced	84 (5.73)	NA	84 (3.79)
	Widowed	13 (0.89)	NA	13 (0.59)
Education, *n* (%)	Junior high school or below	320 (21.81)	685 (91.21)	1005 (45.31)
	High school or secondary technical school	267 (18.20)	62 (8.26)	329 (14.83)
	Associate degree or undergraduate degree	772 (52.62)	4 (0.53)	776 (34.99)
	Master’s degree or above	108 (7.36)	NA	108 (4.87)
Disability ^a^, *n* (%)	None	959 (65.37)	540 (71.90)	1499 (67.58)
	Level 4	63 (4.29)	59 (7.86)	122 (5.50)
	Level 3	50 (3.41)	33 (4.39)	83 (3.74)
	Level 2	164 (11.18)	51 (6.79)	215 (9.69)
	Level 1	231 (15.75)	68 (9.05)	299 (13.48)
Rural/urban, *n* (%)	Rural	266 (18.13)	122 (16.25)	388 (17.49)
	Urban	1201 (81.87)	629 (83.75)	1830 (82.51)
Career, *n* (%)	Unemployed/dropout	406 (27.68)	51 (6.79)	457 (20.60)
	Student	124 (8.45)	650 (86.55)	774 (34.90)
	Employed	823 (56.10)	NA	823 (37.11)
	Retired	114 (7.77)	NA	114 (5.14)
	Preschool child	NA	50 (6.66)	50 (2.25)

*NA* not applicable, *SD* standard deviation.

^a^In China, disability is divided into four levels, in which Level 1 is the most severe and Level 4 is the mildest.

### Family status

As shown in Table 2, most (65.55%) patients had 3–4 family members; however, most patients (70.51%) also indicated that they were the only affected individual in their household. Compared with minor patients, adult patients had fewer family members (*P* < 0.001); however, compared with minor patients, adult patients had more affected individuals at home (*P* < 0.001). The proportion of adult patients who had affected parents or children (17.04%) was significantly greater than that of minor patients (5.86%) (*P* < 0.001). Similarly, the proportion of affected biological siblings (15.68%) was greater among adult patients than among minor patients (4.53%, *P* < 0.001). However, no significant difference was observed in the prevalence of other affected blood relatives between the adult (6.82%) and minor patient groups (6.66%, *P* > 0.05). The percentage of consanguineous marriages among the parents of adult patients (6.95%) was greater than that among the parents of minor patients (1.33%, *P* < 0.001). In terms of family economic status, the annual household income per capita of 77.68% of the patients did not exceed RMB 100,000, and the annual household income per capita of adult patients was less than that of minor patients (*P* < 0.001). In terms of the stability of family income, the self-rated scores of the respondents (2.79 ± 1.11) were slightly greater than the median score, and there was no difference between the adult group (2.76 ± 1.14) and the minor group (2.84 ± 1.05, *P* > 0.05) in this context.

**Table 2:** Family status of the surveyed patients with IRDs.

	Category	Adults	Children	Overall	*P* *
N		1467	751	2218	
Number of family members^a^, *n* (%)	1–2	171 (11.66)	22 (2.93)	193 (8.70)	< 0.001
	3–4	936 (63.80)	518 (68.97)	1454 (65.55)	
	≥ 5	360 (24.54)	211 (28.10)	571 (25.74)	
Number of patients in the family^a^, *n* (%)	1	946 (64.49)	618 (82.29)	1564 (70.51)	< 0.001
	2–3	378 (25.77)	114 (15.18)	492 (22.18)	
	≥ 4	143 (9.75)	19 (2.53)	162 (7.30)	
Whether any parent or child had IRDs, *n* (%)	Yes	250 (17.04)	44 (5.86)	294 (13.26)	< 0.001
	No	1217 (82.96)	707 (94.14)	1924 (86.74)	
Whether siblings had IRDs, *n* (%)	Yes	230 (15.68)	34 (4.53)	264 (11.90)	< 0.001
	No	1237 (84.32)	717 (95.47)	1954 (88.10)	
Whether other blood relatives had IRDs, *n* (%)	Yes	100 (6.82)	50 (6.66)	150 (6.76)	0.888
	No	1367 (93.18)	701 (93.34)	2068 (93.24)	
Whether the parents were consanguineous^b^, *n* (%)	Yes	102 (6.95)	10 (1.33)	112 (5.05)	< 0.001
	No	1355 (92.37)	739 (98.40)	2094 (94.41)	
	Prefer not to say	10 (0.68)	2 (0.27)	12 (0.54)	
Annual household income per capita (RMB), *n* (%)	Less than 50 K	731 (49.83)	309 (41.15)	1040 (46.89)	< 0.001
	50–100 K	439 (29.93)	244 (32.49)	683 (30.79)	
	100–200 K	185 (12.61)	106 (14.11)	291 (13.12)	
	200–300 K	56 (3.82)	45 (5.99)	101 (4.55)	
	More than 300 K	56 (3.82)	47 (6.26)	103 (4.64)	
Self-rated household income stability (0−5), mean ± SD		2.76 ± 1.14	2.84 ± 1.05	2.79 ± 1.11	0.111

*SD*: standard deviation.

^a^ The number of family members and the number of affected individuals at home, including the patient.

^b^ In China, marriage between consanguineous relatives refers to marriage with direct blood relatives or collateral blood relatives within three generations.

* Tests were performed to identify the differences between adult patients and minor patients.

### Symptoms

**Fig. 1:**
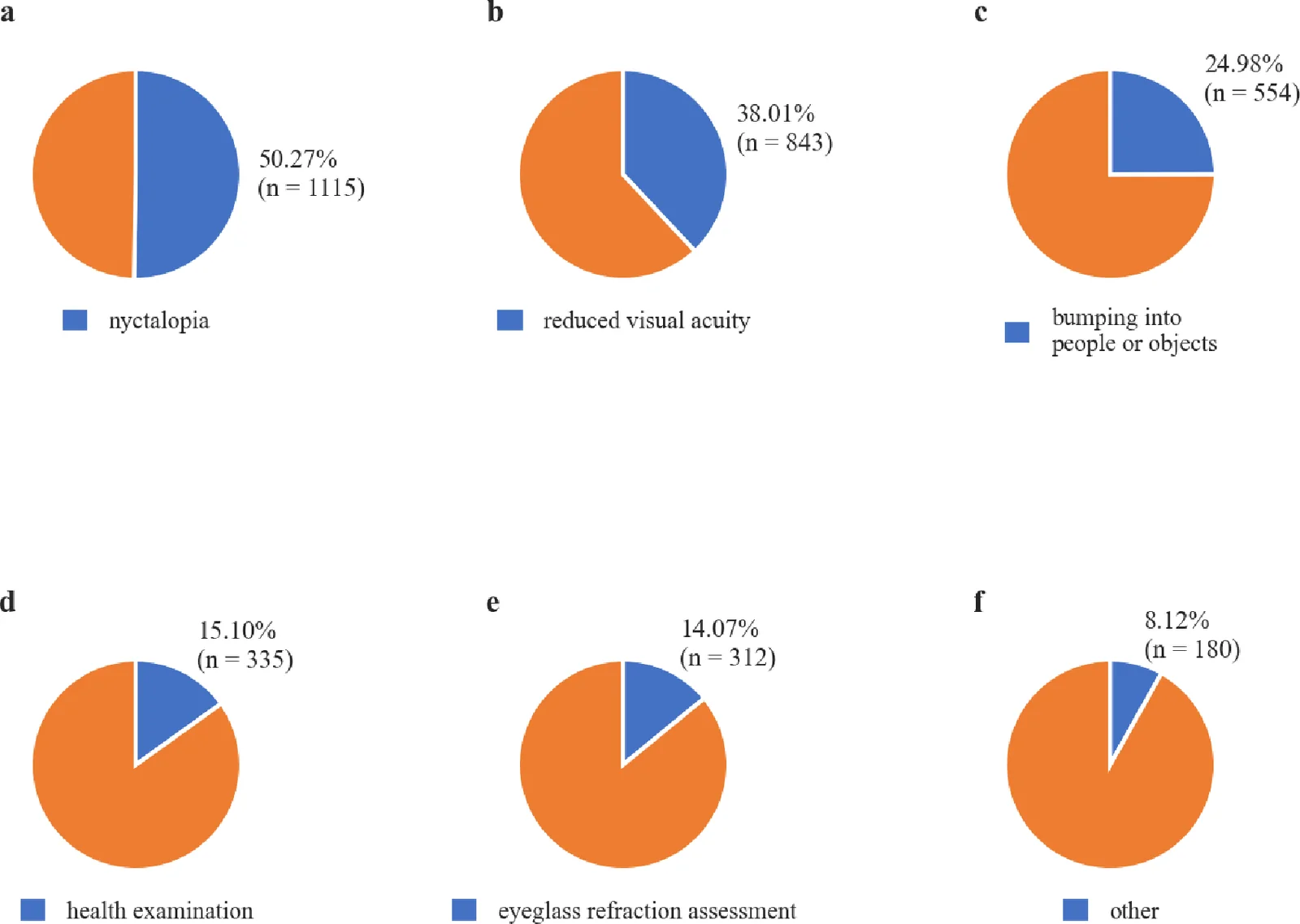
How patients discovered their ocular abnormalities. Six pie charts (**a**–**f**) display the proportions of how patients discovered their ocular abnormalities, including nyctalopia (50.27%), reduced visual acuity (38.01%), bumping into people or objects (24.98%), health examination (15.10%), eyeglass refraction assessment (14.07%), and other (8.12%).

For the sample of patients included in the present study, the age of onset was 13.11 ± 12.69 years; the corresponding figure for the adult group was 17.39 ± 13.50 years, and that for the minor group was 4.74 ± 3.74 years, which indicated a significant difference between the two groups (*P* < 0.001). The ways in which the survey respondents’ ocular abnormalities were discovered are shown in Fig. 1. These discoveries were prompted by nyctalopia (50.27%), reduced visual acuity (38.01%), bumping into people or objects (24.98%), health examinations (15.10%), eyeglass refractions (14.07%), and other channels (8.12%). Because some patients’ ocular abnormalities were discovered through multiple channels (e.g., some patients had nyctalopia and low vision simultaneously), the sum of all the percentages in Fig. 1 does not equal 100%.

This study also investigated whether an inflection point was evident in the changes in the visual function of patients, that is, a time point when visual function changed from relatively stable to a sudden decline or a time point when visual function changed from a slow decline to a rapid decline. In this study, respondents were nearly equally divided between those who believed that such turning points existed and those who did not. A total of 49.91% of the respondents believed that they had not experienced such an inflection point. A total of 50.09% of the respondents believed that they had experienced one or more inflection points. With respect to the question regarding which moments corresponded to the inflection point or which events triggered the inflection point, 24.12% of the respondents chose “the COVID-19 epidemic”, 8.16% chose “overwork”, 5.50% chose “childbirth”, 5.28% chose “intense emotional fluctuations”, 1.49% chose “marriage”, 1.31% chose “serious illness”, 1.08% chose “injury”, and 13.89% chose “other”.

### Diagnosis and treatment

As shown in Table 3, the age of diagnosis of the patients was 18.98 ± 14.53 years, which indicates a mean difference of 5.87 years compared with the age of onset (13.11 ± 12.69 years). The mean age at diagnosis in the adult group was 25.58 ± 13.50 years, which was 8.19 years greater than the mean age at onset (17.39 ± 13.50 years); furthermore, the mean age in the minor group was 6.07 ± 3.97 years, with a mean difference of 1.33 years compared with the age at onset (4.74 ± 3.74 years). In terms of misdiagnosis experience, 42.47% of patients had experienced one or more misdiagnoses, and 6.00% of patients had experienced five or more misdiagnoses. The number of misdiagnoses in the minor group was less than that in the adult group (*P* < 0.001).

**Table 3:** Diagnostic experience of the surveyed patients with IRDs.

	Category	Adults	Children	Overall	*P* *
N		1467	751	2218	
Age at diagnosis, mean ± SD		25.58 ± 13.50	6.07 ± 3.97	18.98 ± 14.53	< 0.001
Number of misdiagnoses, *n* (%)	Never	937 (63.87)	339 (45.14)	1276 (57.53)	< 0.001
	1−2	367 (25.02)	294 (39.15)	661 (29.80)	
	3−4	82 (5.59)	66 (8.79)	148 (6.67)	
	≥ 5	81 (5.52)	52 (6.92)	133 (6.00)	

*SD*: standard deviation.

* Tests were performed to identify the differences between adult patients and minor patients.

**Fig. 2:**
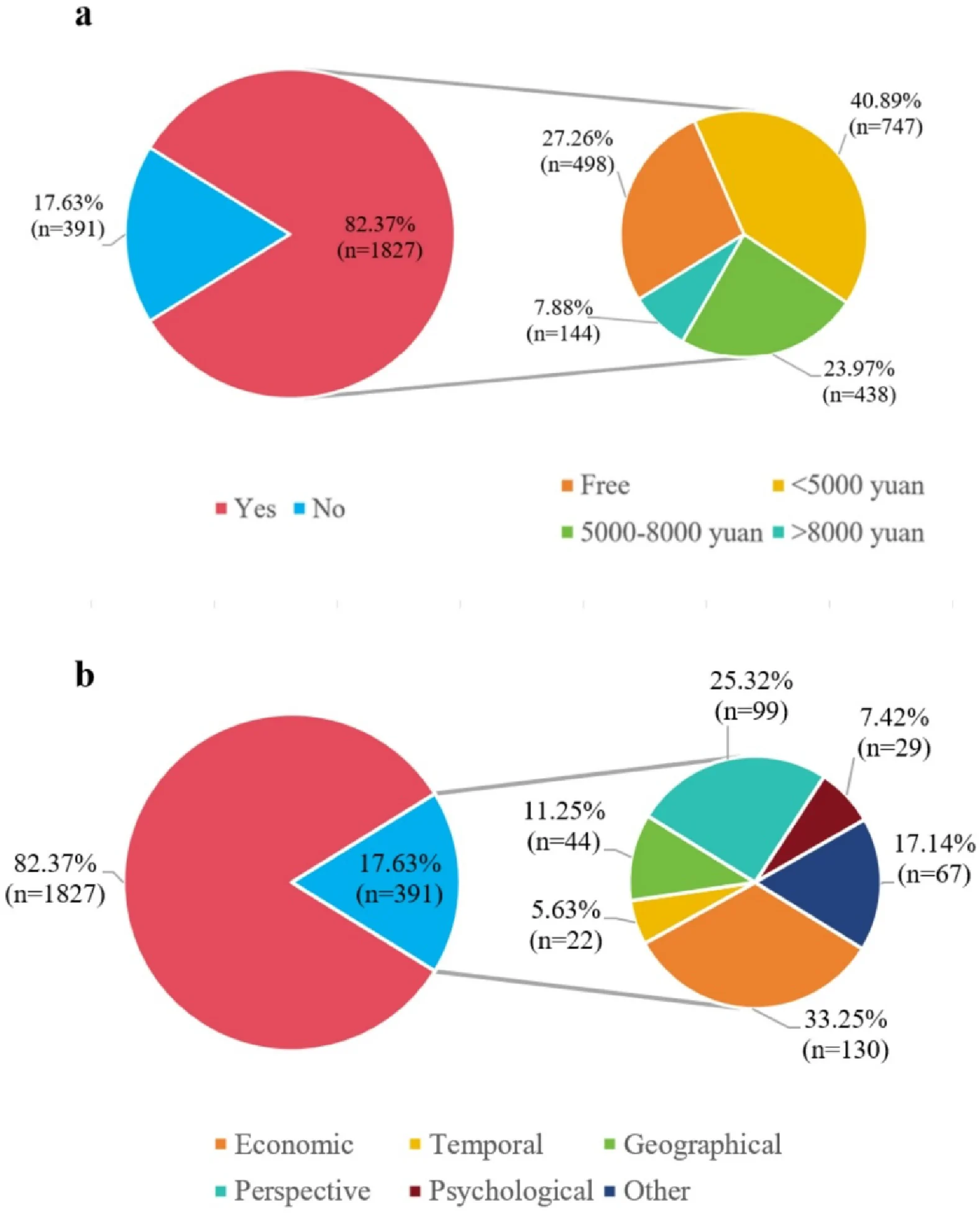
Genetic testing status of the patients. Two pie chart groups (**a**, **b**) show patients’ genetic testing status. (**a**) 82.37% “Yes” (underwent genetic testing), with cost breakdown: <5000 yuan (40.89%), 5000–8000 yuan (23.97%), Free (27.26%), and > 8000 yuan (7.88%). (**b**) 17.63% “No” (did not undergo genetic testing), with primary reason breakdown: Economic (33.25%), Perspective (25.32%), Other (17.14%), Geographical (11.25%), Psychological (7.42%), and Temporal (5.63%).

Since IRDs are genetic diseases and genetic testing is the gold standard for diagnosis, understanding the genetic testing status of patients is necessary. As shown in Figs. 2 and 82.37% of the patients underwent genetic testing, whereas 17.63% of the patients did not undergo genetic testing. Among the patients who underwent genetic testing (Fig. 2a), 27.26% received such testing for free, 40.89% paid less than RMB 5,000, 23.97% paid between 5,000 and 8,000 RMB, and 7.88% paid more than RMB 8,000. The primary reasons for which patients reported not having participated in genetic testing were as follows: 33.25% was attributed to economic factors, 5.63% was attributed to temporal factors, 11.25% was attributed to geographic factors (too far away), 25.32% was attributed to perspective factors (unnecessary test), 7.42% was attributed to psychological factors (such as an inability to confront the test results), and 17.14% was attributed to other reasons (Fig. 2b).

Among the 750 treated patients, 73.47% had received complementary and alternative therapies (traditional Chinese medicine and nutritional supplements), 7.87% had received therapies lacking evidence-based support (e.g., vascular bypass-type surgeries, serum or nerve growth factor injections), 4.27% had received complication management (e.g., cataract surgery, retinal detachment repair, glaucoma surgery), 3.07% had received gene-specific therapies, 2.80% had received cell therapy (e.g., stem cells), 0.93% had received retinal prosthesis treatment, 0.80% had received optogenetic therapy, 0.67% had received off-label medication (e.g., minocycline), and 10.80% had received other treatments (e.g., self-administered eye drops). This was a multiple-choice question, and the proportions across treatment categories sum to more than 100%; among the 1468 patients who did not receive treatment, 66.96% did not receive treatment because of a lack of effective therapy. The cost and treatment effect of patients who received treatment are shown in Table 4. The total treatment cost of 63.33% of the patients was less than RMB 50,000. The total cost of treatment for the adult group was less than that for the minor group (*P* = 0.028). The treatment effect of 70.27% of patients was far from meeting or did not meet expectations; the treatment effect of 26.00% of patients met expectations, and the treatment effect of 3.73% of patients exceeded or far exceeded expectations. In terms of the perception of the treatment effect, there was no significant difference between the adult group and the minor group (*P* = 0.213).

**Table 4:** Cost and effect of treatment for patients.

	Category	Adults	Children	Overall	*P* *
*N*		569	181	750	
Total cost of treatment (RMB), *n* (%)	<50,000	377 (66.26)	98 (54.14)	475 (63.33)	0.028
	50,000−100,000	121 (21.27)	50 (27.62)	171 (22.80)	
	100,000−200,000	50 (8.79)	22 (12.15)	72 (9.60)	
	>200,000	21 (3.69)	11 (6.08)	32 (4.27)	
Treatment effect, *n* (%)	Far from meeting expectations	252 (44.29)	74 (40.88)	326 (43.47)	0.213
	Did not meet expectations	158 (27.77)	43 (23.76)	201 (26.80)	
	Met expectations	137 (24.08)	58 (32.04)	195 (26.00)	
	Exceeded expectations	18 (3.16)	6 (3.31)	24 (3.20)	
	Far exceeded expectations	4 (0.70)	0 (0.00)	4 (0.53)	

* Tests were performed to identify the differences between adult patients and minor patients.

This study also considered the treatment costs that patient families could afford if effective treatment options were available. As shown in Table 5, approximately half (48.78%) of the patients could afford total costs of less than 100,000 yuan, and only 4.55% of patients could afford total costs of more than 1 million yuan. Patients in the minor group could afford higher treatment costs than those in the adult group could (*P* < 0.001). If the payments could be made in installments over 10 years, the expenses that patients could bear increased; 24.93% of patients could afford less than 10,000 yuan a year, and 8.39% could afford more than 100,000 yuan a year. With payment made in installments, the expenses that patients in the minor group could bear were also greater than those that patients in the adult group could bear (*P* < 0.001).

**Table 5:** Treatment costs for effective therapy that are affordable for patient families.

	Category	Adults	Children	Overall	*P* *
*N*		1467	751	2218	
For effective therapy, the total cost that a patient in the family can pay, *n* (%)	<100,000	783 (53.37)	299 (39.81)	1082 (48.78)	< 0.001
	100,000−300,000	438 (29.86)	229 (30.49)	667 (30.07)	
	300,000−600,000	145 (9.88)	111 (14.78)	256 (11.54)	
	600,000−1,000,000	50 (3.41)	62 (8.26)	112 (5.05)	
	>1,000,000	51 (3.48)	50 (6.66)	101 (4.55)	
The amount that a patient can afford each year if the payment can be made in installments over 10 years, *n* (%)	<10,000	416 (28.36)	137 (18.24)	553 (24.93)	< 0.001
	10,000−30,000	569 (38.79)	285 (37.95)	854 (38.50)	
	30,000−60,000	277 (18.88)	158 (21.04)	435 (19.61)	
	60,000−100,000	105 (7.16)	85 (11.32)	190 (8.57)	
	>100,000	100 (6.82)	86 (11.45)	186 (8.39)	

* Tests were performed to identify the differences between adult patients and minor patients.

### Rehabilitation

This study asked patients “If rehabilitation training was offered by public welfare organizations, such as targeted walking training, information acquisition training, and living and vocational skills training, which could indirectly improve your quality of life, would you be willing to participate?” The respondents selected answers on a scale ranging from 0 to 10. The patient’s willingness score to participate in rehabilitation training was 7.75 ± 3.01; the score of the adult group was 7.29 ± 3.20, and the score of the minor group was 8.65 ± 2.34, which significantly differed (*P* < 0.001). Among all the respondents, 113 (5.09%) chose 0, i.e., they had no intention at all; 2105 (94.91%) scored between 1 and 10, i.e., they were more or less willing. This study also asked these 2105 respondents who were willing to participate in rehabilitation training the following question: “If the rehabilitation training was in another city, would you still be willing to participate?” Similarly, on a scale ranging from 0 to 10, patients’ willingness to participate in rehabilitation training was 4.51 ± 3.28, in which the score associated with the adult group was 4.18 ± 3.30 and the score associated with the minor group was 5.12 ± 3.16, indicating a significant difference (*P* < 0.001).

Owing to visual impairment, many patients with IRDs require the use of assistive devices. In the sample in this study, 1,004 patients (45.27%) used one or more assistive devices. The details are shown in Table 6. The assistive devices, listed in descending order of usage frequency, were flashlights (16.82%), magnifying glasses (15.15%), screen reading software (14.07%), vision aids (8.57%), white canes (4.46%), and glasses (2.21%), and 2.25% of patients used other assistive devices. Among the 2218 patients investigated in this study, no guide dogs were used. The use of assistive devices differed between the adult group and the minor group; the proportion of the adult group using screen reader software (*P* < 0.001), flashlights (*P* < 0.001), and white canes (*P* < 0.001) was greater than the corresponding proportion of the minor group, and the proportions associated with the use of magnifying glasses (*P* < 0.001), visual aids (*P* < 0.001), and glasses in the minor group were greater (*P* = 0.004).

**Table 6:** Types of assistive devices used by patients.

	Category	Adults	Children	Overall	*P* *
*N*		1467	751	2218	
Screen reader software	Yes	271 (18.47)	41 (5.46)	312 (14.07)	< 0.001
	No	1196 (81.53)	710 (94.54)	1906 (85.93)	
Magnifying glass	Yes	182 (12.41)	154 (20.51)	336 (15.15)	< 0.001
	No	1285 (87.59)	597 (79.49)	1882 (84.85)	
Vision aids	Yes	62 (4.23)	128 (17.04)	190 (8.57)	< 0.001
	No	1405 (95.77)	623 (82.96)	2028 (91.43)	
Flashlight	Yes	339 (23.11)	34 (4.53)	373 (16.82)	< 0.001
	No	1128 (76.89)	717 (95.47)	1845 (83.18)	
White canes	Yes	92 (6.27)	7 (0.93)	99 (4.46)	< 0.001
	No	1375 (93.73)	744 (99.07)	2119 (95.54)	
Glasses	Yes	23 (1.57)	26 (3.46)	49 (2.21)	0.004
	No	1444 (98.43)	725 (96.54)	2169 (97.79)	
Guide dog	Yes	0 (0.00)	0 (0.00)	0 (0.00)	–
	No	1467 (100.00)	751 (100.00)	2218 (100.00)	
Other assistive devices	Yes	31 (2.11)	19 (2.53)	50 (2.25)	0.531
	No	1436 (97.89)	732 (97.47)	2168 (97.75)	

* Tests were performed to identify the differences between adult patients and minor patients.

### Impact on life

The first thing that is affected by visual function is travel (walking out of the house and going out to participate in activities). In this study, 74.39% of the patients were able to travel independently; 76.41% of the adults and 70.44% of the minors could travel independently (*P* = 0.002). Among the adult patients who could not travel independently, 98.84% were unable to travel because of poor visual function; among the minor patients who could not travel independently, only 45.95% were unable to travel because of poor visual function, and the remaining patients were unable to travel because of age or other reasons. IRDs also affected other aspects of patients’ lives (Table 7). The proportions of patients whose lives were strongly affected in terms of their family, hobbies, career, emotions, self-care, and interpersonal relationships were 67.85%, 59.83%, 59.29%, 57.26%, 54.19%, and 47.16%, respectively. A greater proportion of patients in the minor group chose “no effect” because of age. Therefore, in all the aspects in Table 7, the proportion of patients in the adult group who experienced relatively large or enormous influences was greater than the corresponding proportion of patients in the minor group.

**Table 7:** Effects of IRDs on the lives of patients.

	Category	Adults	Children	Overall	*P* *
*N*		1467	751	2218	
Self-care	No impact	102 (6.95)	320 (42.61)	422 (19.03)	< 0.001
	Less impact	114 (7.77)	33 (4.39)	147 (6.63)	
	Certain impact	335 (22.84)	112 (14.91)	447 (20.15)	
	Relatively large impact	382 (26.04)	117 (15.58)	499 (22.50)	
	Enormous impact	534 (36.40)	169 (22.50)	703 (31.70)	
Career	No impact	119 (8.11)	482 (64.18)	601 (27.10)	< 0.001
	Less impact	63 (4.29)	1 (0.13)	64 (2.89)	
	Certain impact	224 (15.27)	14 (1.86)	238 (10.73)	
	Relatively large impact	356 (24.27)	62 (8.26)	418 (18.85)	
	Enormous impact	705 (48.06)	192 (25.57)	897 (40.44)	
Hobby	No impact	80 (5.45)	228 (30.36)	308 (13.89)	< 0.001
	Less impact	93 (6.34)	28 (3.73)	121 (5.46)	
	Certain impact	306 (20.86)	156 (20.77)	462 (20.83)	
	Relatively large impact	447 (30.47)	151 (20.11)	598 (26.96)	
	Enormous impact	541 (36.88)	188 (25.03)	729 (32.87)	
Emotion	No impact	34 (2.32)	261 (34.75)	295 (13.30)	< 0.001
	Less impact	86 (5.86)	38 (5.06)	124 (5.59)	
	Certain impact	348 (23.72)	181 (24.10)	529 (23.85)	
	Relatively large impact	445 (30.33)	125 (16.64)	570 (25.70)	
	Enormous impact	554 (37.76)	146 (19.44)	700 (31.56)	
Family	No impact	45 (3.07)	124 (16.51)	169 (7.62)	< 0.001
	Less impact	89 (6.07)	13 (1.73)	102 (4.60)	
	Certain impact	325 (22.15)	117 (15.58)	442 (19.93)	
	Relatively large impact	442 (30.13)	168 (22.37)	610 (27.50)	
	Enormous impact	566 (38.58)	329 (43.81)	895 (40.35)	
Interpersonal relationships	No impact	113 (7.70)	305 (40.61)	418 (18.85)	< 0.001
	Less impact	151 (10.29)	41 (5.46)	192 (8.66)	
	Certain impact	407 (27.74)	155 (20.64)	562 (25.34)	
	Relatively large impact	385 (26.24)	114 (15.18)	499 (22.50)	
	Enormous impact	411 (28.02)	136 (18.11)	547 (24.66)	

* Tests were performed to identify the differences between adult patients and minor patients.

## Discussion

The sample in this study included more male patients with IRDs than female patients with IRDs; the male-to-female ratio was approximately 6:4, and the proportion of males in the minor group (63.52%) was greater than that in the adult group (58.42%), which differs from the situation in the United States. A study conducted in the U.S. with a large sample size revealed that males and females were affected equally by IRDs^23^. Among adult patients (≥ 18 years), nearly one-third (32.72%) were never married, which is significantly higher than the rate of being never married among the Chinese population older than 15^24^. The proportion of patients with IRDs who held disability certificates (32.42%) was much greater than that of the general population (approximately 3%), and the proportion of adults (34.63%) who had disability certificates was greater than that of minors (28.10%). The progressive nature of IRDs explains the higher proportion of adults with disability certificates. Approximately 37% of the disabled population in China has a disability certificate^25^. Many patients with IRDs do not apply for disability certificates. Therefore, the actual proportion of patients with IRDs who have disabilities may be much greater than the proportion of patients with certificates. In addition, this study revealed that the unemployment rate among patients with IRDs was relatively high, which was consistent with the findings reported by other studies^11,14^.

In terms of family status, adult patients had more affected individuals in their families; namely, 35.51% of adult patients had other affected individuals in their families, whereas only 17.71% of minor patients had other affected individuals in their families. This finding may be because symptoms become more obvious as the disease progresses with age and the number of other affected individuals in the adult patient’s family increases over time. In addition, the percentage of consanguineous marriages among the parents of adult patients (6.95%) was greater than that among the parents of minor patients (1.33%). Given that more than one-third of the world’s population is heterozygous for at least one recessive variant that leads to IRDs^26^, consanguineous marriages greatly increase the risk of children developing IRDs, and this difference may be expected to reduce the number of IRD patients in China in the future. In terms of economic status, the annual income per capita of 77.68% of the patients’ families did not exceed 100,000 yuan, and the self-rating of income stability was not high, thus indicating that IRDs had a negative impact on the economic income of patient families.

The mean age at IRD onset was 13.11 years. Symptoms were discovered based on the following opportunities: nyctalopia (50.27%), reduced visual acuity (38.01%), bumping into people or objects (24.98%), health examinations (15.10%), eyeglass refractions (14.07%), and other channels (8.12%). These data provide a foundation for educating the public on how to identify IRDs earlier. This study also revealed that approximately half of the respondents believed that they had experienced an inflection point with respect to the development of symptoms. These inflection points were almost all related to major changes in physical and/or emotional status. These findings suggest that in the health management of patients with IRDs, measures must be taken to avoid excessive emotional and physical changes.

The average age at diagnosis of IRD patients was 18.98 years, which was 5.87 years older than the average age at onset, thus highlighting the difficulty of diagnosis. The average duration from onset to diagnosis was 8.19 years for adult patients but only 1.33 years for minor patients on average. This difference is similar to that of other rare diseases (e.g., Dai et al.^27^), reflecting a trend toward improving the overall diagnosis of rare diseases in China. Although most Chinese patients with IRDs were diagnosed at the first visit, 42.47% of patients still experienced one or more misdiagnoses, and 6.00% of patients even experienced five or more misdiagnoses. China’s National Health Commission released two editions of guidelines for the diagnosis and treatment of rare diseases in 2019 and 2025^28,29^, including the diagnosis and differential diagnosis methods for 207 rare diseases, including IRDs such as retinitis pigmentosa, Leber congenital amaurosis, and choroideremia. The release of these two editions of diagnosis and treatment guidelines will contribute to reducing the number of misdiagnoses of rare diseases, including IRDs. Genetic testing is key for the diagnosis of the etiology of IRDs and provides key information for accurate treatment and family genetic risk assessment^30^. In this study, 82.37% of the patients underwent genetic testing, whereas 17.63% of the patients did not undergo genetic testing. The two most important reasons for the lack of participation in genetic testing were economic (no money for the test) and perspective (the belief that the test was not needed) factors. For economic reasons, the government can increase subsidies; for perspective reasons, on the one hand, disease education can be strengthened, and on the other hand, treatment methods must be developed to make patients understand the necessity of testing.

Owing to the lack of effective treatments, only approximately one-third (33.81%) of the patients received treatment, of whom 73.47% received complementary and alternative therapies. Patients were generally dissatisfied with the effect of treatment; specifically, 70.27% of patients who received treatment believed that the effect did not meet or was far from meeting their expectations. These findings are related to insufficient social investment in the research and development of IRD treatments. Although the burden of IRDs on patients and society is similar to that of some common diseases, societal investment in IRD research is significantly lower. For example, in 2019, Parkinson’s disease caused economic losses of 53.8 billion USD in the U.S. and received 224 million USD in research funding, whereas IRDs caused economic losses in the U.S. between 13.4 billion USD and 31.8 billion USD but received only 11 million USD in research funding^17^. Even if effective treatment options are developed in the future, the families of patients will continue to face financial constraints. In this study, approximately half (48.78%) of the patients could afford the total cost of effective treatment options of less than RMB 100,000. If the payment could be made in installments over 10 years, the treatment costs that patients could bear were higher. Many countries have designed financial security policies for IRD patients. For example, France provides personalized autonomy allowances, disability compensation benefits, aid from the national housing agency and financial aid for visual technical aids for individuals with vision impairment^31^. Although China provides economic security measures such as living assistance, employment support, medical and rehabilitation assistance, and public transportation discounts for individuals with vision impairment, the strength and implementation of these measures must still be strengthened.

A Canadian survey revealed that 50% of IRD patients did not use support resources such as mobility training and vocational rehabilitation; the most common reason for this lack of use was that they did not believe that they needed such resources^18^. However, rehabilitation training can improve the quality of life of patients with IRDs^32–34^. This study revealed that patients with IRDs in China have a relatively strong willingness to participate in rehabilitation training and that the willingness of minor patients was greater than that of adult patients. This finding reflects the fact that in Chinese family culture, parents want their children to have a better life. However, if the rehabilitation training is located in another city, the patient’s willingness to participate is reduced, which may be related to the inconvenience of patient travel and the travel expenses incurred in traveling to other places. Service providers should attempt to provide rehabilitation training for patients locally as much as possible, consider the possibility of remote training or further study the reasons why patients are unwilling to go to other cities and provide targeted solutions. During the recovery process, the use of assistive devices can improve the convenience of patients’ daily lives. An Australian study revealed that only 2% of patients who were legally blind because of IRDs did not use any assistive devices^35^. However, in our study, as many as 54.73% of patients did not use assistive devices. This situation shows that there is still much room for promoting the use of assistive devices among IRD patients in China.

IRDs have a significant negative effect on a patient’s ability to travel. A total of 74.39% of the patients included in this research could still travel independently. However, even patients who can walk independently may face difficulty walking in unfamiliar or dark environments, as well as challenges in going up and down stairs and escalators and using public transportation^5^. The visual impairment of patients and the resulting impairment of movement have negative effects on many aspects of their lives. IRDs had relatively strong effects or strong effects on the lives of more than half or nearly half of the patients in terms of family, hobbies, careers, emotions, self-care, and interpersonal relationships. These findings were consistent with those of previous studies. For example, family members may not understand the patient’s progressive vision loss, which can cause the patient to feel nervous^5^. In terms of hobbies, nyctalopia makes it difficult for patients to go to certain restaurants and theaters with dim lighting^36^, and they may also be unable to continue hobbies such as reading and sports^10,11^. In terms of career, many patients are frustrated in job hunting because of vision problems or can perform only some specific jobs or have to change jobs frequently to adapt to their constantly decreasing working ability^5,15^. In terms of emotions, many studies^15,37,38^ have shown that patients with IRDs suffer from high levels of psychological stress because of a variety of emotional problems, and patients in different age groups are affected. In terms of self-care, patients exhibit an impaired ability to perform basic daily tasks and live independently^31^. In terms of interpersonal relationships, many patients feel ignored, discriminated against, or not understood^18,39^.

### Limitations

First, this study did not verify respondents’ diagnoses. The inclusion criterion required respondents to report that the patient had been diagnosed with an IRD by a medical institution, but this information was not independently verified. The names and subtypes of IRDs are complex, and patients may misrecall or misunderstand their diagnoses; 17.63% of respondents had not undergone genetic testing (the diagnostic gold standard for IRDs). Diagnostic misclassification is therefore possible, and the relevant data should be interpreted with caution. Second, the present study relied primarily on an online survey. Although we conducted a telephone survey of 20 participants who were unable to answer the questions online, sample bias resulting from the use of the online survey still cannot be ruled out. Since this study focused on patients with IRDs and impaired visual function, sample bias from online surveys may be more prominent. Third, as a cross-sectional survey, the present study was unable to observe the dynamic changes in the patient’s condition, nor could it provide a detailed description of the mechanisms underlying certain important findings (e.g., how IRDs affect the career of patients). These questions are expected to be answered by future longitudinal and qualitative studies. Fourth, treatment types were surveyed with a multiple-choice question, but per-type costs were not collected, and treatment outcome was evaluated with a single global question; costs and outcomes therefore cannot be stratified by treatment type.

## Conclusion

This study described in detail the general features, family status, symptoms, diagnosis and treatment, rehabilitation, and impact of IRDs on the lives of patients in China. In general, the condition of these patients was poor in all aspects, which had serious negative effects on many aspects of their lives. The government must invest more research and development (R&D) funds in the treatment and rehabilitation of patients with IRDs and formulate and improve social security policies for IRD patients to help reduce the financial and psychological burden faced by these patients and improve their quality of life.

## Data Availability

The datasets used and/or analysed during the current study available from the corresponding author on reasonable request.

## References

[1] Georgiou, M., Fujinami, K. & Michaelides, M. Inherited retinal diseases: therapeutics, clinical trials and end points—a review. *Clin. Exp. Ophthalmol.* **49**, 270–288. 10.1111/ceo.13917 (2021). 33686777

[2] Schneider, N. et al. Inherited retinal diseases: linking genes, disease-causing variants, and relevant therapeutic modalities. *Prog Retin Eye Res.* **89**, 101029. 10.1016/j.preteyeres.2021.101029 (2022). 34839010

[3] Tatour, Y. & Ben-Yosef, T. Syndromic inherited retinal diseases: genetic, clinical and diagnostic aspects. *Diagnostics* **10**, 779. 10.3390/diagnostics10100779 (2020). 33023209 PMC7600643

[4] Ben-Yosef, T. Inherited retinal diseases. *Int. J. Mol. Sci.* **23**, 13467. 10.3390/ijms232113467 (2022). 36362249 PMC9654499

[5] Senthil, M. P., Khadka, J. & Pesudovs, K. Seeing through their eyes: lived experiences of people with retinitis pigmentosa. *Eye* **31**, 741–748. 10.1038/eye.2016.315 (2017). 28085147 PMC5437327

[6] Das, K. et al. Factors influencing the choice of low-vision devices for visual rehabilitation in Stargardt disease. *Clin. Exp. Optom.* **102**, 426–433. 10.1111/cxo.12867 (2019). 30582217

[7] Wu, K. Y. et al. Retinitis pigmentosa: novel therapeutic targets and drug development. *Pharmaceutics* **15**, 685. 10.3390/pharmaceutics15020685 (2023). 36840007 PMC9963330

[8] Chew, L. A. & Iannaccone, A. Gene-agnostic approaches to treating inherited retinal degenerations. *Front. Cell. Dev. Biol.* **11**, 1177838. 10.3389/fcell.2023.1177838 (2023). 37123404 PMC10133473

[9] Chan, H. W., Oh, J. & Leroy, B. Therapeutic landscape for inherited ocular diseases: current and emerging therapies. *Singap. Med. J.* **64**, 17–26. 10.4103/singaporemedj.smj-2022-179 (2023). PMC9979805 36722513

[10] Chivers, M. et al. The burden of X-linked retinitis pigmentosa on patients and society: a narrative literature review. *Clin. Outcomes Res.* **13**, 565–572. 10.2147/ceor.s297287 (2021). PMC8236258 34188501

[11] Garip, G. & Kamal, A. Systematic review and meta-synthesis of coping with retinitis pigmentosa: implications for improving quality of life. *BMC Ophthalmol.* **19**, 181. 10.1186/s12886-019-1169-z (2019). 31409325 PMC6693213

[12] Humphries, A. et al. Quality of life analysis in patients with retinitis pigmentosa. *Ophthalmic Res.* **67**, 348–357. 10.1159/000539116 (2024). 38718781

[13] Ng, Q. X., Chan, H. W., Lim, R. B. T. & Koh, G. C. H. This is life: An interpretative phenomenological analysis of the lived experience of working-age adults with inherited retinal diseases in Singapore. *Disabil. Health J.* **18**, 101819. 10.1016/j.dhjo.2025.101819 (2025). 40044541

[14] Galvin, O. et al. The impact of inherited retinal diseases in the Republic of Ireland (ROI) and the United Kingdom (UK) from a cost-of-illness perspective. *Clin. Ophthalmol.* **14**, 707–719. 10.2147/opth.s241928 (2020). 32184557 PMC7062501

[15] Watanabe, K., Aouadj, C., Hiratsuka, Y., Yamamoto, S. & Murakami, A. Quality of life and economic impacts of retinitis pigmentosa on Japanese patients: a non-interventional cross-sectional study. *Adv. Ther.* **40**, 2375–2393. 10.1007/s12325-023-02446-9 (2023). 36947329 PMC10032244

[16] Chay, J. et al. The economic burden of inherited retinal disease in Singapore: a prevalence-based cost-of-illness study. *Eye* **37**, 3827–3833. 10.1038/s41433-023-02624-7 (2023). 37301937 PMC10698171

[17] Gong, J. et al. The impact of inherited retinal diseases in the United States of America (US) and Canada from a cost-of-illness perspective. *Clin. Ophthalmol.* **15**, 2855–2866. 10.2147/opth.s313719 (2021). 34234408 PMC8257071

[18] Kherani, I. Z., Andrews, C., Pereira, J. A., Moniz, L. S. & Qian, C. X. Impact of inherited retinal diseases on Canadian patients and families: a mixed-methods study. *Can. J. Ophthalmol.* **58**, 532–538. 10.1016/j.jcjo.2022.06.021 (2023). 35905942

[19] Pernice, H. F. et al. Patient journey with Charcot-Marie-Tooth Disease—a German patient survey study. *Orphanet J. Rare Dis.* **21**, 84. 10.1186/s13023-026-04236-2 (2026). 41634720 PMC12958679

[20] Galesic, M. & Bosnjak, M. Effects of questionnaire length on participation and indicators of response quality in a web survey. *Public. Opin. Q.* **73**, 349–360. 10.1093/poq/nfp031 (2009).

[21] Ministry of Civil Affairs of the People’s Republic of China. Press conference on promoting high-quality development: new achievements in civil affairs work [Press conference transcript]. State Council Information Office. (2024). https://english.scio.gov.cn/m/pressroom/2024-11/11/content_117539190.html

[22] National Bureau of Statistics of China. Statistical communiqué of the People’s Republic of China on the 2024 national economic and social development. (2025). http://www.stats.gov.cn/english/PressRelease/202502/t20250228_1958822.html. Accessed 13 Aug 2025.

[23] Abbass, N. J. et al. Trends and disparities in the incidence and prevalence of inherited retinal diseases in the United States. *Am. J. Ophthalmol.* **279**, 165–173. 10.1016/j.ajo.2025.07.021 (2025). 40706695

[24] National Bureau of Statistics of China. Marital status of the population aged 15 and above [Table data] . (2023). https://data.stats.gov.cn/easyquery.htm?cn=C01&zb=A030608&sj=2024. Accessed 13 Aug 2025

[25] China Disabled Persons’ Federation. Number of persons with disabilities and their classification by type and disability level in China. (2021). https://www.cdpf.org.cn/zwgk/zccx/cjrgk/15e9ac67d7124f3fb4a23b7e2ac739aa.htm

[26] Hanany, M., Rivolta, C. & Sharon, D. Worldwide carrier frequency and genetic prevalence of autosomal recessive inherited retinal diseases. *Proc. Natl. Acad. Sci. U S A*. **117**, 2710–2716. 10.1073/pnas.1913179117 (2020). 31964843 PMC7007541

[27] Dai, S. et al. A cross-sectional survey on the health status of patients with Charcot-Marie-Tooth disease in a Chinese national patient group. *J. Neurol.* **272**, 322. 10.1007/s00415-025-13063-7 (2025). 40198420

[28] National Health Commission of the People’s Republic of China. Rare disease diagnosis and treatment guidelines. https://www.nhc.gov.cn/wjw/c100175/201902/9fe60f6aa3b141fca4e76ba1745c37f2.shtml (2019). Accessed 13 Aug 2025.

[29] National Health Commission of the People’s Republic of China. Diagnosis and treatment guidelines for 86 rare diseases. https://www.nhc.gov.cn/yzygj/c100068/202507/5b3f41180a42465eb9eec34597bacaf2.shtml (2025). Accessed 13 Aug 2025.

[30] Lam, B. L. et al. Genetic testing and diagnosis of inherited retinal diseases. *Orphanet J. Rare Dis.* **16**, 514. 10.1186/s13023-021-02145-0 (2021). 34906171 PMC8670140

[31] Cross, N., van Steen, C., Zegaoui, Y., Satherley, A. & Angelillo, L. Retinitis pigmentosa: burden of disease and current unmet needs. *Clin. Ophthalmol.* **16**, 1993–2010. 10.2147/opth.s365486 (2022). 35757022 PMC9232096

[32] Chu, H. Y. & Chan, H. S. The effect of vocational training on visually impaired people’s quality of life. *Healthcare* **12**, 692. 10.3390/healthcare12060692 (2024). 38540657 PMC10970319

[33] Colombo, L. et al. Managing retinitis pigmentosa: a literature review of current non-surgical approaches. *J. Clin. Med.* **14**, 330. 10.3390/jcm14020330 (2025). 40283384 PMC12027910

[34] Kamali, N. & Ashori, M. The effectiveness of orientation and mobility training on the quality of life for students who are blind in Iran. *Br. J. Vis. Impair*. **41**, 108–120. 10.1177/02646196211019066 (2023).

[35] Jin, R., Petoe, M. A., McCarthy, C. D., McGinley, J. L. & Ayton, L. N. Perspectives on traditional and emerging mobility aids amongst Australians with inherited retinal disease. *Br. J. Vis. Impair*. **43**, 527–539. 10.1177/02646196241253514 (2025).

[36] Jangra, D. et al. Psychosocial adjustment to visual loss in patients with retinitis pigmentosa. *Ophthalmic Genet.* **28**, 25–30. 10.1080/13816810701201930 (2007). 17454744

[37] Garcia, G. et al. Profound vision loss impairs psychological well-being in young and middle-aged individuals. *Clin. Ophthalmol.* **11**, 417–427. 10.2147/opth.s113414 (2017). 28260855 PMC5328297

[38] Redgrave, S. & McNeill, A. A qualitative interview study of the attitudes toward reproductive options of people with genetic visual loss. *J. Genet. Couns.* **31**, 1231–1234. 10.1002/jgc4.1601 (2022). 35781904 PMC9796805

[39] Bakir, M. et al. Navigating a hidden disability: lived experiences and challenges of adults with early stage inherited retinal diseases. Disabil. *Health J.* **18**, 101820. 10.1016/j.dhjo.2025.101820 (2025). 40055042

